# The Industrial Diseases: Their Importance and Methods of Study

**Published:** 1913-08

**Authors:** W. Gilman Thompson

**Affiliations:** Professor of Medicine, Cornell University Medical College in New York City


### BUFFALO MEDICAL JOURNAL

VOLUME 69 AUGUST, 1913 No. 1

### ORIGINAL ARTICLES

*No responsibility is assumed by the Journal for the opinions of contributors of original articles; nor for methods of expression nor errors in proof reading, excepting for articles in a foreign language contributed for translation. The right is reserved to decline contributions on account of bona fide lack of space; or because they do not bear on practical medicine and surgery; or because they would fail to interest or would give offense to the majority of subscribers. The expression of minority views or reasonable and courteous criticism of the views of others is not considered offensive.*

### The Industrial Diseases: Their Importance and Methods of Study

BY W. GILMAN THOMPSON, M.D.
Professor of Medicine, Cornell University Medical College in New York City

[A paper read before the Academies of Medicine in Buffalo and in Rochester, April 8th and 9th, 1913]

THE industrial development of this country has been so rapid, so varied and extensive, that few persons have realized the degree to which concomitant diseases have arisen as a result of industrial hazards. These diseases may be defined as resulting: (1) from contact with harmful materials, (2) from harmful methods of work, (3) from harmful environment. An illustration of the first group would be poisoning by inhaling wood alcohol fumes or phosphorous fumes; of the second group would be the locomotive engineer's sciatica, due to sitting sidewise on a bench, subjecting the right hip to constant pressure and jolting; of the third group, would be compressed air illness from working in a caisson, where not the materials used, nor the mode of handling them constitute the hazards, but the environment of increased atmospheric pressure.

It is natural that the industrial accidents should first have received attention, for in many cases the relationship of cause and effect are immediate and obvious even to the layman. But the industrial diseases are, for the most part, of insidious and chronic development, and when acute their symptoms may be so unfamiliar as not to be attributed to their true cause, even by physicians. For instance, I have known a fatal acute case of wood alcohol poisoning in a man employed to varnish the interior of a closed beer vat to be diagnosed as death from "epilepsy."

Comparatively few persons are aware that a man may acquire tremors, paralysis and a premature senile dementia from making felt hats, because they are dipped in a nitrate of mercury solution. Nor do they know that drop wrist may result from pottery glazing, or that both jaws may be lost from making white phosphorus matches, or that the septum of the nose may become perforate from making chrome pigments. Yet all these are not casual but almost certain dangers under peculiar conditions of work, conditions which, with proper knowledge and enforcement of hygienic precautions, are easily remediable.

Although a number of isolated studies of industrial diseases have from time to time appeared in the medical literature of this country, and a few more have been made by state health departments, labor bureaus and factory commissions, it is only very recently that the subject has begun to excite widespread interest or that investigations have been begun upon a comprehensive scale. In June, 1910, the first American National Conference upon Industrial Diseases was convened in Chicago, and in a memorial addressed by it to the President, it was stated that there occur annually in the United States, thirteen million four hundred thousand cases of sickness among artisans and craftsmen, many of which are attributed to occupation hazards, involving a total annual economic loss of nearly three-fourths of a billion dollars. It is high time, therefore, that physicians bestir themselves to look carefully into the problems involved and aid in gathering accurate data, in order clearly to differentiate the influence of occupational environment and disease hazard in special lines of work.

The second meeting of this conference took place in conjunction with the last meeting of the American Medical Association in June, 1912, and for the first time the latter organization devoted considerable discussion to the industrial diseases. In September of last year the International Congress of Applied Chemistry, meeting in Washington, for the first time also devoted a session to the subject of industrial poisons.

In foreign countries, on the other hand, notably in England, Germany, France and Belgium, the industrial diseases have long been systematically studied and the idea that Employers' Liability Acts should cover pecuniary responsibility for diseases as well as injuries acquired through dangerous trades, has been incorporated into statutes. This idea is rapidly extending through this country, and as a basis of legislation it is realized that authoritative statistics should be obtained, hence the enactment of the physicians' notification law, for specified industrial diseases occurring in this State, which went into effect in September,

1911. This law was copied almost verbatim from a similar notification act of Great Britain, although the latter now comprises 28 industrial diseases instead of the six which we are required to report in New York. Eight other States, namely, California, Michigan, Wisconsin, Connecticut, Illinois, New Jersey, Maryland and Minnesota, have lately fallen into line and passed a similar law to that of New York, requiring report in some cases to State Labor Bureaus, in others to State Health Departments.

It is extremely desirable that physicians should offer earnest co-operation in this matter of reporting, for the subject of industrial diseases is of pressing importance not only from the point of view of medical science but as well from its humanitarian, economic and legislative aspects. Industrial *accidents* can be diagnosed and reported in many cases by laymen, but the physician is usually the only one who can determine the nature and extent of industrial *diseases*, and rightly apportion the influence, for example, of a metal poison in producing arteriosclerosis or nephritis, as compared with such coincident factors as alcoholism and syphilis. It is a great mistake to rush hastily into remedial legislation until we possess more definite knowledge of the prevalence and seriousness of the diseases caused by industry.

Up to the present time, in the United States, legislation, with only one or two exceptions, has taken the form, as far as industrial diseases are concerned, of attempting to regulate factory ventilation, a problem which is of extreme difficulty owing to the lack of universally accepted standards. About twenty states have vague laws requiring that factories shall be "well ventilated" or "sufficiently ventilated," and ten states specify a minimum cubic air space per occupant. New York State is the only one which provides for systematic analysis of factory air with publication of the results, and Illinois has made good progress in maintaining compulsory standards of air purity. With the exception of these two states, together with New Jersey and Massachusetts, there has been little or no scientific factory inspection in the whole country, designed specifically to control industrial diseases. In the states mentioned, however, some very valuable intensive studies have been made by official inspectors of special industries, notably of the lead, pottery and pearl button industries, as well as those involving the use of mercury and phosphorus.

Although statistics of the industrial diseases are as yet very meagre in this country, I am able to present a sufficient number to be convincing that the subject amply merits all the attention which is being given to it, and I will refer briefly to a few of the more important industrial disease hazards. There are about 150

distinct occupations in which lead poisoning is prevalent, and 27 in which arsenic constitutes the hazard, although the substitution of analine dyes has largely replaced the use of the latter substance in the coloring of wall papers and artificial flowers.

Dr. John B. Andrews, in *Bulletin*, 95, of the State Bureau of Labor, reported 60 fatal cases of lead poisoning occuring in New York State in 1909-10. Three more fatal cases recently occurred in a single smelting establishment in New York City.

Dr. E. E. Pratt, investigating for the Committee of the Labor Legislation Association, recently found eighteen cases of lead poisoning among men employed in the Brooklyn Navy Yard to scrape red paint from the hulls of the battleships. Three of these men came to my clinic with lead palsy.

In my service in the Presbyterian and Bellevue Hospitals and the Cornell University Medical College Dispensary, I have collected the histories of over 300 cases of serious plumbism observed during the past eight years, 75 per cent. of which were among painters. In most of these cases there was total incapacity for work, lasting for months or years. Some of these patients had complete paralysis of the hands, many had lead colic, and most had arterio-sclerosis. Some acquired chronic Bright's disease and practically all suffered from anemia, digestive disorders and muscular weakness. One youth of 23 years had been employed for eight years in a paint manufactory as a helper. He had the hardened arteries of the octagenarian, a greatly enlarged heart, diseased kidneys and dyspnoea.

A bill designed to mitigate lead poisoning, particularly in the pottery industry, was introduced recently in the New Jersey Legislature. Having passed the Assembly it was blocked in the Senate by the statements of a representative of some of the lead industries, but in the very town in which this man lives, the records of ninety-four cases of lead colic, drop wrist, etc., have since been taken from the records of a single physician.

Dr. Alice Hamilton, in a report on the white and red lead industry for the United States Department of Labor, found examples of lead poisoning in thirty-three of fifty-six establishments where lead was used in process of manufacture, with a yearly average of 665 cases. It is a very striking fact that in England, in the white lead works near Newcastle, compulsory medical inspection has so far reduced the cases of lead poisoning that in 1910 the ratio was one case among every 264 employees, whereas in Illinois, without legalized inspection and control, the ratio was one case among every seven employees.

Mercury poisoning was formerly common among the makers of mirrors, but the use of mercury has largely been replaced by silver in this process. In making felt hats, however, the felt is dipped into a preservative solution of nitrate of mercury, and subsequently in the process of hat pressing the mercury is volatilized and may be inhaled by the workmen or women.

Mrs. Lindon W. Bates, in a report made for the Women's Welfare Department of the National Civic Federation of New York, studied 102 cases of chronic mercurial poisoning. The fumes of the metal give rise to loosening of the teeth, ulcers of the mouth, diseases of the jaw bones, anemia, weakness and serious digestive disturbances. In extreme cases tremors and dementia may occur, as above cited. In a powder works in which fulminate of mercury ($Hg2\ CNO$) is prepared, I have lately seen a number of cases of superficial ulceration of the fingers and arms of the workmen, due to the corrosive action of the mercury powder upon the skin. Mercury poisoning is also common among makers of incandescent lamps.

Some time ago a man came to my Clinic showing a perforating ulcer between the two nasal cavities, large enough to admit the forefinger, which was the result of chromic acid chronic poisoning. He had also suffered from double vision, nausea, vomiting and difficulty in fixing the attention. On his hands were round depressed scars of old ulcers or "chrome holes," as the workmen term them. The patient, who was a chemist, had found chromic acid in the abundant nasal mucus and in his urine. There were forty workmen employed in the chrome works with this patient, all but four of whom had chronic inflammation in the nose, and half of them had perforation of the nasal septum. A boy employed in the works had recently died in a sanitarium having violent vomiting and a yellow skin, the results of chrome poisoning.

Phosphorus poisoning acquired in the manufacture of matches, where white phosphorus is used, has been the subject of an admirable study by Dr. John B. Andrews. The result of this poison, although the cases are numerically few, as compared with those of poisoning in many trades, are more disfiguring than those of any other substance, for the phosphorus fumes entering the mouth cause decay of the teeth, and rapidly progressive ulceration and destruction of the jaw bones, which must be removed, in whole or in part, to save the victim's life. Dr. Andrews found records of forty cases of necrosis of the jaws in a single factory, "of which fifteen resulted in the loss of one or both jaws and several cases resulted in death." In another small factory were records of twenty-one cases, six of which developed in a single year. Fortunately the next Congressional legislation will in future abolish this hazard by taxing the use of white phosphorus out of existence.

Wood alcohol, often used as a solvent in varnish, if inhaled in concentrated form, gives rise to acute poisoning, causing permanent blindness and grave disturbances of the circulation which may occasion death by paralysis of the heart. A number of fatal cases have occurred in New York City, and recently two men who went down into a large beer vat in a brewery in Buffalo, to varnish it, were overcome by the vapor of wood alcohol, with the result that one died and the other became permanently blind.

Professor Baskerville of the College of the city of New York, told me lately that he has in press a study of industrial wood alcohol poisoning in which he has collected from the literature upwards of 1,000 cases.

Brass founder's ague is a fairly common industrial disease, and is at present being made the subject of an intensive study by the Committee on Occupational Diseases of the New York Branch of the American Association for Labor Legislation.

The making of pottery offers many hazards to the workmen. Those employed in mixing the damp clay suffer from bronchitis and joint pains. Those employed in applying the glaze may acquire lead poisoning, and the grinders and polishers inhale dust which may contain silica, alumina, charcoal and flint which are highly irritant to the respiratory mucous membranes. giving rise to chronic bronchitis and pneumokoniosis. Such victims often subsequently acquire pulmonary tuberculosis, which is graphically called "potter's rot" among them.

One of the most interesting of the industrial diseases due to environment is largely increasing. I refer to the compressed air illness. Originally confined to tunnel workers, it is now also met with among those employed in sinking deep caissons for the foundations of bridge piers, sky-scrapers, etc.

A member of my staff, Dr. Edward L. Keays, has published the most exhaustive report extant upon this subject, comprising the results of his experience while employed as physician to the tubes of the Pennsylvania Railroad under the East River in New York City. Among the 10,000 workmen employed, he studied 3,692 cases, twenty of which were fatal.

In my medical clinic in Bellevue Hospital and the Cornell Medical College Dispensary, so largely frequented by laborers and artisans, a special study of the industrial diseases has been conducted for several years and a great variety is met with, from the neurasthenia of the garment maker to the chronic pulmonary diseases of those employed in dusty trades, the thoracic and other deformities of faulty positions while at work, the chronic nephritis, arteriosclerosis and cardiac hypertrophy of the metal poisons, the skin lesions of dyers, the disorders produced by inhalation of toxic fumes, the caisson disease and many other diseases resulting in more or less serious disability and sometimes in death.

A scientific classification of the industrial or occupational diseases becomes a necessity at the outset of their study, yet such classification presents considerable difficulty owing to the vagueness of existing nomenclature, the over-lapping of disease hazards in the different industries and the subdivisions of labor in the individual industry. This is illustrated in the above example of the pottery trade, where the hazard may be due to lead, to dust inhalation, or the environment of exposure to damp and cold. Again, in the felt hat industry, there are about twenty different occupations in the employment, in only two or three of which mercury constitutes the hazard. Obviously, to class a man's occupation as "hatter" means very little from the point of view of an industrial disease, for he may either sell hats over a bargain counter, make straw hats, in which there is no special hazard, or be a "coner" or "presser" of felt and die from mercury poisoning. It is, therefore, very necessary in reporting occupational diseases to give exact detail as to the particular subdivision under the occupation. In a complete study of this subject it is desirable to have a general classification under three major headings, comprising, namely, (1) the harmful occupation, (2) the harmful substance or harmful environment, and (3) the disease produced. I see no advantage in attempting to draw a rigid distinction between an "industrial" and an "occupational" disease as is sometimes done. The primary meaning of the word industrial is "diligence." One speaks of the lead "industry," rather than lead "occupation," but of the "occupation" of painter, etc. All industrial diseases are occupational, but the reverse is less apparent. One speaks of the occupation of telegrapher giving telegrapher's cramp rather than of the industry of telegrapher. The point is, that it is futile to construct artificial boundaries where so many trades, industries or occupations are ill defined. It is of much more value to adopt a uniform nomenclature and classification so that statistics gathered in one state may be compared at a glance with those from another. The National Census Bureau in conjunction with a Committee of the American Medical Association is at work at present upon this problem, which is far more intricate than might at first thought be supposed. For example, to refer again to the illustration of "hatter." Not only may the workman be employed in making hats of many materials, but as a maker of felt hats he may be blocker, blower, pouncer, flanger, curler, shearer, stiffener, singer, trimmer, coner, dyer, dryer, feeder, hardener, mixer, welter, or finisher! The solution of the problem should be met as follows: By stating the general name of the occupation accurately, as, for example, "felt hat maker," and in parenthesis the specialized work under the general occupation, as "coner," together with the hazardous substance used, which in this instance is mercury. Thus those unfamiliar with the subject may easily learn the special hazards of particular work and be on the lookout for them. This has been done to some extent by Sir Thomas Oliver and in the classification which I furnished the New York State Labor Bureau for publication in the booklet of information on the occupational diseases issued to physicians on request. One more example under this system would read as follows:

1. Occupation: potter.
2. Specialized work: polisher.
3. Injurious substances: silica, flint, charcoal and lead dust.
4. Disease: chronic bronchitis, asthma, fibroid phthisis, lead neuritis or palsy, arteriosclerosis, interstitial nephritis.

I have emphasized this matter because incomplete classification is puzzling and may be useless. For example, in one of the Census Bulletins of 1910 a list of 101 occupations is given by name in which there may be disease hazard, but what the disease may be is not always apparent. For instance, in this list the caisson disease (although required for notification under the several state laws), is not mentioned, but the making of neckties is stated, and I have thus far been unable to find wherein the special hazard consists for this trade.

To facilitate the collection and grouping of data regarding industrial diseases I make use, in my Clinic, of special history blanks which are quickly filled up.

Whatever classification is used, it should be both comprehensive and elastic, for new disease hazards are constantly arising with the rapid advances in manufacturing industries and all kinds of material employments. Thus the handling of ferrosilicon and smelting of vanadium as well as cyanid processes, all recently used in the steel industry, have furnished serious and sometimes fatal instances of poisoning. A friend who is occupied with wireless telegraphy and telephoning invention told me that among employees using high frequency electric currents who are constantly exposed to ozone inhalations, the bronchial irritation has proved so extreme that special ventilation appliances have been found necessary to mitigate the risk.

In conclusion I submit the following general classification as a frame-work under which the occupational disease hazards may be placed in detail as occasion arises:

### General Classification of Occupational Diseases and Harmful Substances.

*A. Harmful Substances*—

1. Toxic metals and their compounds.
2. Toxic gases, vapors and fumes.
3. Toxic fluids (acids, alkalies, dyes, etc.)
4. Irritant dusts and fibres.
   - (a) Insoluble inorganic dusts.
   - (b) Soluble inorganic dusts.
   - (c) Organic dusts and fibres.
5. Organic germs (anthrax, glanders, etc.)
6. Miscellaneous irritants (anilin, tar, fats, etc.)

*B. Harmful Environments*—

1. Air compression, rarefaction and concussion.
2. Excessive humidity.
3. Excessive heat and cold.
4. Excessive light (electric, X-ray, etc.)

*C. Occupational Injuries*—

(Medical)

1. Injuries to nerves, muscles and bones. (Strain, fatigue, cramp, faulty positions, "occupational neuroses," blows, vibrations, pressure, etc.)
2. Injuries to the eyes.
3. Injuries to the ears.
4. Injuries to the nose, throat and mouth.
5. Injuries to the skin.

*Occupational Diseases of the*

1. Blood.
2. Circulatory system.
3. Respiratory system.
4. Nervous system.
5. Digestive system.
6. Muscular system and bones.
7. Cutaneous system.
8. Urinary system.
9. Special sense organs.
10. Associated diseases, tuberculosis, etc.

In order to promote interest in the study of the most important subject of the occupational diseases, I would suggest the formation in the larger cities of committees of those whose interests meet on at least the common ground of statistics. I may be permitted to refer to the personnel of such a committee which I organized two years ago in New York City, and which was subsequently adopted by the local branch of the American Association for Labor Legislation as one of its special committees. It comprises a professor of chemistry, an insurance actuary, an expert statistician, representatives of the American Association for Labor Legislation of the State Labor Bureau and State Bureau of Factory Inspection, a professor of economics, a sociologist and several physicians chosen for their special interest in tuberculosis, the nomenclature and classification of disease, and the clinical aspects of the occupational diseases. This, I believe, forms an ideal basis of organization for study of the subject, and our committee working in close co-operation with the State authorities has in the short time since its organization, done much educational and reformatory work.

---

A Study of Thirty-Nine Cases as Regards the Intestinal Length and Nutrition. L. T. Swaim, Cambridge, Mass., concludes: 1. Ptosis is present probably in many more cases than we realize, 9 out of 29.

2. The size of the stomach varies enormously and the tubular stretched stomach is present in most of the cases of ptosis—6 out of 9—and that the lesser curvature can be greatly stretched down, this series shows.

3. The small intestines vary greatly in length, 10 ft. 6 in. to 25 ft. 10 in., averaging 19 ft. 3 in., the length having only slight effect on the nutrition, with a tendency to more fat the longer the intestine and vice versa.

4. The large intestines vary also, averaging 5 ft. 3 in., extremes 3 ft. 8 in. to 8 ft. 5 in.

When the stomach is low the large intestine is apt to be longer and the cecum large and pendulous, the greatest length being in the transverse colon and sigmoid.

5. The total average length of the entire intestines is 24 ft. 6 in.

6. A rather significant fact is that not only the hollow viscera are displaced, but even more often the solid, such as the liver, which was below the costal border in 20 cases.—*Boston Medical and Surgical Journal, August* 29, 1912.

